# Comparison of Machine Learning Models for Colon Cancer Survival: Predictive Modeling Approach

**DOI:** 10.2196/72665

**Published:** 2025-11-26

**Authors:** Reuben Adatorwovor, Motolani E Ogunsanya, Bin Huang, Richard Charnigo, Olufunmilola Abraham

**Affiliations:** 1 Department of Biostatistics, College of Public Health, University of Kentucky, 760 Rose street, Suite 208H, Lexington, KY, 40536, United States, 1 859-218-0959; 2 TSET Health Promotion Research Center, Department of Family and Preventive Medicine, University of Oklahoma Health Sciences Center, Oklahoma City, OK, , United States; 3 Department of Internal Medicine, College of Medicine, University of Kentucky, Lexington, KY, United States; 4 Dr. Bing Zhang Department of Statistics, College of Arts and Sciences, University of Kentucky, Lexington, KY, United States; 5 Department of Pharmacy Practice and Science, College of Pharmacy, University of Kentucky, Lexington, KY, United States

**Keywords:** colon cancer survival, colorectal cancer, Cox model, elastic net, LASSO, machine learning models, random survival forests, risk factors, survival estimation, least absolute shrinkage and selection operator

## Abstract

**Background:**

Colon cancer is a leading cause of cancer-related deaths worldwide, with survival influenced by risk factors, treatment type, and patient characteristics. Traditional statistical models, such as Kaplan-Meier curves, have been widely used to estimate survival probabilities. However, these models often have difficulty handling complex interactions, covariates, and nonlinear relationships between risk factors. Recently, machine learning (ML) techniques have emerged as promising tools for improving survival prediction by handling large covariates and capturing complex patterns.

**Objective:**

This study compares several ML models to accurately estimate colon cancer survival by leveraging data from the Kentucky Cancer Registry. By identifying key risk factors, these analyses aim to improve risk stratification, treatment planning, and prognosis for overall colon cancer survival within subgroups.

**Methods:**

We conducted a retrospective analysis of colon cancer cases diagnosed between 2010 and 2022 (n=33,825), using Kentucky Cancer Registry data linked to mortality records, with approval from the University of Kentucky Institutional Review Board (#63067). We compared multiple predictive modeling techniques, including Cox proportional hazards, accelerated failure time models, Extreme Gradient Boosting, random survival forests, least absolute shrinkage and selection operator (LASSO), and elastic net regression, to estimate survival probabilities. The Kaplan-Meier method provided baseline survival estimates, and multivariate models, including ML approaches, evaluated contributions of key risk factors. Model performance was compared across evaluation metrics such as the Brier score, concordance index, out-of-bag error, and Continuous Ranked Probability Score. Missing data were handled via multiple imputation, and leave-one-out cross-validation was applied to reduce overfitting.

**Results:**

The ML models identified key covariates influencing survival outcomes, such as age, treatment type, positive nodes, tumor stage, smoking, and comorbidities. In the overall model, patients who refused or received no treatment had a 3.24-fold higher risk of mortality compared to those who underwent surgery at primary and regional sites. Elevated mortality risk was also observed among smokers (24% higher than non-smokers) and Appalachian residents (7% higher than non-Appalachian residents). Our overall model achieved a concordance index of 0.8146, with strong discriminatory performance across subgroups, including early-age diagnosis (0.8175), late-age diagnosis (0.7841), Appalachia (0.8135), non-Appalachia (0.8126), White patients (0.8164), and Black patients (0.7881). The results highlight the strengths and limitations of each ML approach, with the random survival forest and LASSO models outperforming traditional methods such as the Cox model in prediction accuracy and model discrimination.

**Conclusions:**

Our study demonstrated the utility of ML in identifying risk factors associated with colon cancer survival, with positive lymph nodes, age at diagnosis, treatment received, clinical tumor size, tumor grade, smoking status, geographic region, and marital status emerging as dominant predictors across all statistical models. This comparative analysis offers valuable insights for clinical decision-making and prognosis, highlighting the potential of ML to identify risk factors specific to different subgroups, ultimately advancing personalized care for patients with colon cancer.

## Introduction

Colon cancer, also known as colorectal cancer (CRC), is a leading cause of cancer-related deaths globally [1,2], typically developing from adenomatous polyps in the colon or rectum. While advancements in screening, early detection, and treatment have improved survival rates from about 50% in the 1970s to approximately 64% today [3], CRC remains a significant public health concern because there are still many deaths and because of survival disparities influenced by stage at diagnosis, the presence of metastasis, and various individual and biological risk factors [1]. Early-stage detection offers better survival outcomes (5 y survival rate of ~90% for localized cases vs ~14% for late-stage diagnoses) [4], yet disparities persist across subgroups, particularly among non-Hispanic Black patients, driven by factors such as socioeconomic status and health care access [3]. Understanding risk factors, both generally and specific to demographic and regional subgroups, is relevant to informing interventions, both broad-based and targeted, for better survival outcomes. Specifically, between 2016 and 2020, Kentucky’s CRC mortality rate was 16.2 per 100,000, compared to the national rate of 13.1 [5]. Thus, in Kentucky, survival rates are notably lower than the national average. The state’s overall 5-year survival rate for colon cancer is approximately 61% [3]. Indeed, Kentucky has one of the highest CRC mortality rates in the United States, which is partially due to high rates of smoking, obesity, and physical inactivity, all of which are modifiable risk factors [4]. Limited access to health care and early screening programs, particularly in rural or Appalachian regions, contributes to delayed diagnoses and poorer survival outcomes [4,6]. Although Kentucky has implemented various cancer control initiatives, such as increasing access to screenings, these efforts have been less effective in rural areas, where health care resources remain scarce [7,8]. As a result, more patients in Kentucky are diagnosed at later stages compared to those in other states, which significantly impacts their chances of survival [9,10].

Colon cancer is influenced by both nonmodifiable and modifiable risk factors. Age is one of the most significant nonmodifiable risk factors, with most cases diagnosed in individuals aged 60 years and above due to accumulated genetic mutations and cellular damage over time [11,12]. A family history of CRC, particularly in first-degree relatives (parents, siblings, or children), implies a higher risk [13], as do inherited genetic syndromes, such as familial adenomatous polyposis and Lynch syndrome, which involve mutations in mismatch repair genes [14]. Among modifiable risk factors, smoking and alcohol use elevate CRC risk through DNA damage and carcinogenic metabolites like acetaldehyde [15,16], with combined use posing an even greater threat [17,18]. Physical inactivity, obesity, and chronic inflammatory conditions like Crohn disease and ulcerative colitis also heighten risk [19]. Regular exercise is associated with reduced CRC risk, by enhancing gastrointestinal motility, reducing systemic inflammation, and supporting a healthy body weight [19,20]. Abdominal obesity increases CRC risk [21] via visceral fat, which triggers chronic inflammation and hormone dysregulation (involving, eg, insulin and leptin) [21,22], particularly for proximal colon cancers [23]. Dietary habits, hormonal factors (especially in women), and screening practices also impact CRC risk, with early detection through colonoscopy, sigmoidoscopy, or fecal occult blood tests significantly improving survival rates [24,25].

The prognosis for patients with colon cancer largely depends on the cancer’s stage at diagnosis, as defined by the TNM staging system [26], which classifies cancer based on tumor size (T), lymph node involvement (N), and the presence of metastasis (M). Patients with stages I and II typically have localized disease, with no lymph node involvement and high survival rates (90%‐95%) when treated early [27]. In stage III, cancer has spread to nearby lymph nodes (but not to distant organs), leading to moderate survival rates (40%‐70%), depending on treatment effectiveness [27]. Stage IV involves distant metastasis, commonly to liver or lungs, resulting in significantly lower survival rates (10%‐15%) [28]. Nonetheless, newer treatment approaches such as targeted therapies, immunotherapies, and palliative care have improved survival and quality of life for patients with metastatic disease [29-31]. Colon cancer continues to be a major global health challenge, underscoring the importance of accurate survival estimation to guide clinical treatment decision-making and public health strategies [32]. Survival outcomes are influenced by early detection, timely intervention, and modifiable lifestyle factors such as diet, exercise, and smoking cessation, although genetic predispositions and medical conditions like inflammatory bowel disease further complicate individual risk profiles. Early screening continues to be the most effective strategy for improving colon cancer survival outcomes.

In recent years, numerous studies [33,34] have applied ML techniques to predict outcomes in colon cancer, demonstrating promising performance in risk stratification and prognosis. However, many of these studies focus on a single model or dataset, limiting generalizability and offering limited comparison across model types using standardized metrics [35]. Additionally, interpretability and the role of geographically linked social determinants, such as residence in medically underserved regions like Appalachia, are often underexplored [10]. This study addresses these gaps by systematically comparing traditional and modern ML approaches including Cox proportional hazards, accelerated failure time (AFT) models, Extreme Gradient Boosting (XGBoost), random survival forests (RSFs), least absolute shrinkage and selection operator (LASSO), and elastic net regression using a large, population-based dataset. By incorporating region-specific variables and evaluating models with consistent performance criteria, this study offers novel insights into both predictive accuracy and the complex risk factors influencing colon cancer survival.

Accurately predicting survival outcomes based on individual risk factors remains a major challenge [36-38], despite significant progress in cancer surveillance and treatment. Understanding how these risk factors influence colon cancer survival is critical for enhancing the effectiveness of surveillance efforts and guiding targeted public health interventions. However, cancer survival surveillance using registry data is often complicated by incomplete or unreliable cause-of-death information, which can undermine the precision of public health interventions and cancer control strategies [39-41]. In this study, we explore the influence of risk factors on prognostic outcomes in colon cancer survival using ML models. Specifically, we seek to answer 2 key questions: (1) how do different risk factors impact cancer survival across various population subgroups? (2) which risk factors emerge as the strongest predictors of survival outcomes within these subgroups?

## Methods

### Data Source and Cohort Selection

The Kentucky Cancer Registry (KCR), a part of the Surveillance, Epidemiology, and End Results (SEER) Program and the National Program of Cancer Registries, provides comprehensive, population-based data on cancer incidence, survival, treatment, and patient demographic factors in Kentucky. KCR routinely links cancer incidence data to Kentucky state mortality data and National Death Index data to ensure updated mortality information. This study included all 33,825 noninstitutionalized adult patients (aged ≥18 y) diagnosed with colon cancer during the study period. Patients with missing diagnosis, treatment, or staging data were excluded, ensuring data integrity.

### Ethical Considerations

This retrospective study utilized deidentified case-level colon cancer data from the KCR for the period January 1, 2010, to December 31, 2022. The study was approved by the University of Kentucky Institutional Review Board (#63067) following the completion of the data use agreement. Because the data were fully anonymized prior to analysis, informed consent was waived. Participant privacy and confidentiality were ensured through strict deidentification procedures, in compliance with institutional policies and federal regulations governing human participant research.

### Patient, Disease, and Treatment Factors

Potential risk factors were selected based on a review of existing literature on colon cancer survival and input from oncology and epidemiology experts on our research team. Clinically relevant variables extracted from the KCR included patient demographic and risk factors such as age at diagnosis, sex, race, ethnicity, marital status, smoking status, cigarette pack years, and geographical residence (Appalachian or non-Appalachian regions of Kentucky). Age at diagnosis was categorized into 2 groups: early onset (18‐60 y) and late onset (over 60 y). Due to the low representation of certain groups, patients who were neither White nor Black were categorized as “Other.” Likewise, ethnicity was classified into “Not of Spanish or Latino origin” and “Other.” Marital status was grouped into broader categories, with divorced and separated individuals combined into a single “Divorced or Separated” category, and those living with a partner or having unknown marital status grouped under “Unknown.”

Histological types [25] not matching “8140” (Adenocarcinoma NOS), “8210” (Papillary Adenocarcinoma, Papillary subtype), “8240” (Tubulovillous Adenocarcinoma), and “8480” (Mucinous Adenocarcinoma) were categorized as “Other/Unspecified.” Treatment records were similarly streamlined based on the first course of treatment: patients reporting no treatment, unknown treatment, or refusal were grouped under “No/Unknown or Refused Treatment.” Those undergoing surgery for primary or regional sites were listed as “Surgery (Primary and Regional),” while individuals receiving multimodal treatment with surgery, chemotherapy, or radiation belonged to “Surgery+(Chemotherapy and/or Radiation).” Therapies not included in the categories described above were classified as “Other.”

Colon cancer staging is often performed using either the American Joint Committee on Cancer (AJCC) TNM system or the SEER Summary Stage system. The AJCC TNM staging is detailed, providing specific breakdowns of the tumor’s size, regional lymph node involvement, and presence of distant metastasis [42]. However, we chose to rely on the SEER 2018 Summary Stage, a simplified system that categorizes cancer’s spread into broader groups: in situ (confined to the origin site), localized (confined to the organ of origin without spread), regional (spread to nearby tissues or lymph nodes), distant (spread to distant organs), and unknown (extent of spread cannot be determined). While the AJCC system offers a more granular, detailed assessment of cancer progression, the SEER Summary Stage aggregates these details into more general categories and is more consistent across years, making it useful for large-scale epidemiological studies and population-based cancer survival tracking [43].

Insurance coverage was reclassified into 4 primary categories to streamline analysis. Patients with private or employer-based insurance were grouped under “Private Insurance.” Those covered by government-funded programs, including Medicare, Medicaid, TRICARE (Military), Workers’ Compensation, Veterans Affairs, and the Indian Health Service, were classified as “Government-Related Programs.” Patients who were uninsured or self-paid were categorized as “Uninsured or Self-Pay,” while individuals with nonspecific, unknown, or unreported insurance status were assigned to the “Other/Unknown Payers” group.

### Statistical Analysis

Descriptive analyses were conducted to summarize patient and clinical characteristics of the colon cancer cohort. Continuous variables, such as age, were reported as means, medians, standard deviations, minimum, and maximum values. Categorical variables, including sex, tumor stage, treatment status, and comorbidity indicators, were summarized using frequency counts and percentages. The descriptive analysis provided an overview of the colon cancer study population cohort and served as the foundation for subsequent survival modeling. They also facilitated the identification of demographic and clinical patterns and covariate distributions relevant to prognosis and interpretation of survival differences across patient subgroups. The results are presented in Table 1.

**Table 1.** Distribution of colon cancer characteristics (continuous variables)^a^.

Risk factors	Frequency, n	Mean (SD)	Median (IQR)	Minimum	Maximum
Age (y)	33,825	65.95 (13.74)	67.00	18.00	103.00
Cigarette pack years	18,602	16.76 (27.29)	0.00	0.00	300.00
Tumor size clinical (mm)	7094	49.04 (36.74)	46.00	0.00	989.00
CS^b^ lymph nodes	19,932	72.72 (125.04)	0.00	0.00	800.00

a Analyses are based on a retrospective cohort study of patients with colon cancer using data from the Kentucky Cancer Registry collected between January 1, 2010, and December 31, 2022.

b CS: clinical stage.

Classical survival analysis methods, such as the Kaplan-Meier estimator [44] and the Cox proportional hazards model [45], have long been the standard approach for analyzing time-to-event data due to their interpretability, capacity for coping with right censoring, and well-established statistical foundations. Yet, they may not be well suited to high-dimensional data. The Kaplan-Meier estimator relies on stratification. If only a few strata are specified, then the results may be subject to confounding or may obscure interactions. If many strata are specified, then the results may be limited by small stratum sizes or may be subject to concerns about multiple comparisons. In contrast, ML methods like XGBoost, RSFs, LASSO, and elastic net Cox regression [1] models offer greater flexibility. RSFs can capture interactions and nonlinear relationships without assuming proportional hazards, while elastic net provides variable selection and regularization in high-dimensional settings. AFT models, though parametric, can serve as a bridge between classical and ML approaches by directly modeling survival time.

We now outline our key statistical approaches for identifying risk factors associated with colon cancer survival (from time of diagnosis until death or right censoring) in Kentucky adults. We begin by summarizing the colon cancer data from the KCR. We constructed nonparametric Kaplan-Meier curves [44] to evaluate differences in survival distributions across subgroups and to explore associations between individual risk factors and survival. To assess the joint impact of risk factors, we used multivariable models, employing both classical and ML approaches. Classical methods included the Cox proportional hazards model [45] and the AFT model [46], which rely on specific assumptions such as proportional hazards (for Cox) or predefined survival time (for AFT) distributions. We also used the ML methods of XGBoost [47], RSF [48], LASSO regression [49], and elastic net [50], which offer greater flexibility.

Subgroup analyses were also conducted to identify influential predictors of survival outcomes within strata based on race (White, Black, and other patient subgroups), time of diagnosis (early onset: 18‐60 y vs late onset: over 60 y), and geographic region (Appalachian vs non-Appalachian Kentucky). These stratified analyses allowed us to assess whether the impacts of key risk factors differed across demographic and regional subgroups so that subgroup-specific predictors of colon cancer survival might be identified.

The multivariate Cox proportional hazards model is a semiparametric statistical method used in cancer survival studies and allows for the simultaneous analysis of multiple risk factors, estimating the hazard of death while controlling for confounders. This approach is beneficial in identifying independent risk factors impacting survival, while adjusting for the effects of other covariates. Additionally, the model enables the investigation of differences between subgroups, providing a more comprehensive understanding of the factors affecting survival outcomes.

The AFT model is an alternative to the Cox proportional hazards model that is useful when the proportional hazards assumption is not met or when focusing on modeling time to an event rather than hazards. The AFT model assumes that covariates accelerate or decelerate survival time, which is useful in assessing how risk factors can shorten or extend a patient’s survival time. Unlike the Cox model’s focus on hazard ratios, the AFT model directly interprets time, offering clearer insights into absolute survival durations, an essential factor in understanding patient prognoses.

XGBoost is a powerful and scalable ML algorithm widely used for structured data. Built on the framework of gradient-boosted decision trees, it is highly effective in tasks such as classification, regression, and survival analysis. Its popularity stems from its ability to handle large datasets efficiently and reduce overfitting through regularization methods. Additionally, XGBoost supports parallel and distributed computing, which speeds up model training and accommodates missing data.

The RSF is a nonparametric ML technique that extends the traditional random forest algorithm to survival data. In our study, the RSF model was utilized to predict patient outcomes based on extensive risk factors. Its major advantage lies in its ability to capture interactions and nonlinear relationships between risk factors, dynamics that traditional models like Cox or AFT might miss. Moreover, RSF’s capacity to handle censored data and missing values makes it robust for real-world clinical datasets. The model also aids in feature selection by identifying the strongest predictor variables, helping clinicians focus on key risk factors that drive survival outcomes.

The LASSO and the elastic net regularization techniques were utilized for the colon cancer survival data because the number of risk factors was relatively large. Regularization is a method of constraining or penalizing model parameters to prevent overfitting, leading to better model fit and generalization.

### LASSO Regression

LASSO is a regularized version of linear regression, where the model is penalized using the $L_{1}norm$ (sum of the absolute values of the coefficients). The goal of using LASSO is to improve the prediction accuracy and interpretability of the model by forcing some of the coefficients that do not contribute to predicting survival to exactly 0. This essentially is equivalent to performing feature selection. The mathematical formulation of the LASSO is as follows:


$$\hat{\beta_{lasso}}=\;\begin{array}{c} argmin\\ \beta \end{array}\;\left(\;\sum_{i=1}^{n}{\left(y_{i}-\;X_{i}^{T}\beta \right)}^{2}+\lambda \sum_{j=1}^{p}|\beta_{j}|\right)$$

Here, $y_{i}$ is the observed target or censored time for colon cancer death in an $i^{th}$ individual, $X_{i}$ is the vector of risk factors, $\beta_{j}$ is the coefficient of the $j^{th}$ predictor or risk factor, λ is the regularization parameter that controls the strength of penalty, and *p* is the number of parameters.

### Elastic Net

Elastic net is a regularization technique that combines both $L_{1}$ regularization (LASSO) and $L_{2}$ regularization (Ridge). It was introduced to overcome some of the limitations of LASSO, particularly when dealing with highly correlated risk factors. Elastic net performs better in situations where there are many predictors, some of which may be correlated. The mathematical formulation of elastic net is as follows:


$$\hat{\beta_{elastic\;net}}=\;\begin{array}{c} argmin\\ \beta \end{array}\;\left(\;\sum_{i=1}^{n}{\left(y_{i}-\;X_{i}^{T}\beta \right)}^{2}+\lambda_{1}\sum_{j=1}^{p}|\beta_{j}|+\lambda_{2}\sum_{j=1}^{p}\beta_{j}^{2}\right)$$

Here, $\lambda_{1}$ and $\lambda_{2}$ are the regularization hyperparameters corresponding to $L_{1}$ and $L_{2}$ penalties, respectively.

The LASSO procedure was chosen because we believe that only a subset of the predictors is most relevant for colon cancer survival prognosis. LASSO is particularly effective for sparse data, where many features are present, but only a few are significant, and its automatic feature selection enhances model interpretability. Elastic net was selected because the risk factors may be correlated with each other, and elastic net is better suited for handling multicollinearity.

### Model Performance

**Figure 1.**
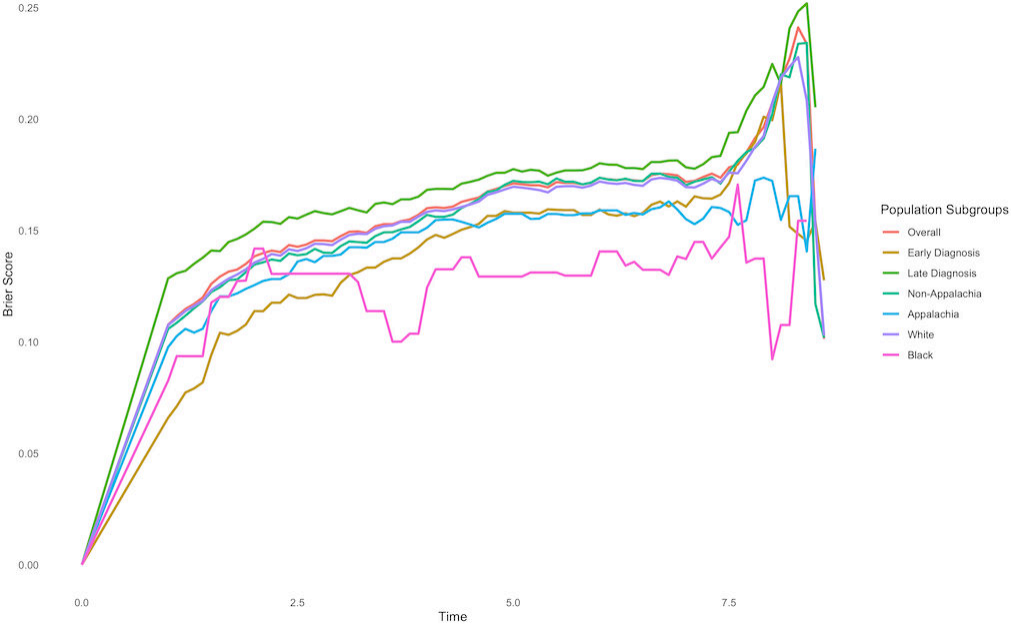
Model performance based on Brier scores for the Cox model. Analyses are based on a retrospective cohort study of patients with colon cancer using data from the Kentucky Cancer Registry collected between January 1, 2010, and December 31, 2022.

Model performance was evaluated using the Brier score (Figures1  2), standardized out-of-bag (OOB) Continuous Ranked Probability Score (CRPS), and concordance index (C-index; see the *Result* section) to assess both calibration and discrimination across the overall cohort and predefined subgroups. The Brier score evaluates the accuracy of predicted probabilities by measuring the mean squared error between predictions and outcomes, with strengths in calibration and sharpness. The CRPS extends the Brier score to continuous outcomes and, when standardized and computed using OOB samples, offers an unbiased performance estimate that helps guard against overfitting. The C-index measures a model’s ability to correctly rank outcomes, making it useful for survival or ranking tasks. Multiple imputation was used to address missing data, and leave-one-out cross-validation was employed to reduce overfitting and ensure robust performance estimates. All hypothesis tests were performed at the 5% significance level. All statistical analyses were conducted using R statistical software [51] or Python [52]. The reporting of this study conforms to STROBE (Strengthening the Reporting of Observational Studies in Epidemiology) and TRIPOD+AI ( Transparent Reporting of a Multivariable Prediction Model for Individual Prognosis or Diagnosis Using Artificial Intelligence) guidelines [53-55].

**Figure 2.**
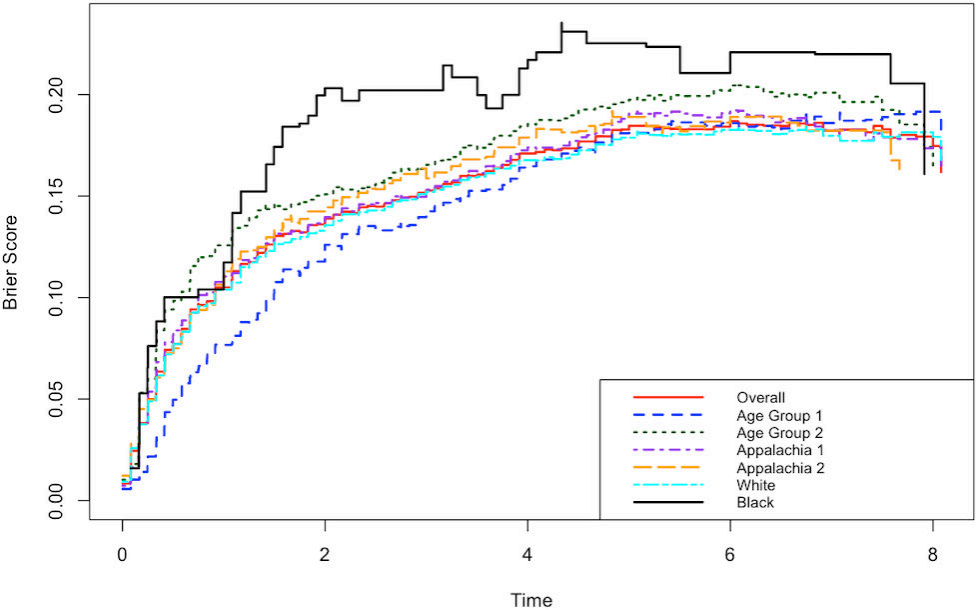
Random survival forest (RSF) model fit assessed via Brier scores by subgroups. Analyses are based on a retrospective cohort study of patients with colon cancer using data from the Kentucky Cancer Registry collected between January 1, 2010, and December 31, 2022.

## Results

### Overview

The results from a total sample of 33,825 including 15,838 patients who were alive and censored as of December 31, 2022, are shown below. The following risk factors for colon cancer survival were investigated: age at diagnosis, treatment, sex, race, ethnicity, marital status, smoking status, cigarette pack years, tumor grade, positive lymph nodes, clinical tumor size, geographic region (Appalachian status), histology classification or type, clinical stage (CS) lymph nodes, insurance, and SEER 2018 summary classification of the tumor. Tables1  2 show the distribution of the risk factors, while Tables 3 and 4 (Tables S1 and S2 in Multimedia Appendix 1) display or compare findings from the various methods.

**Table 2.** Distribution of colon cancer characteristics (categorical variables)^a^.

Risk factors	Count, n (%)
Sex	
Male	17,740 (53.0)
Female	15,730 (47.0)
Race	
White patients	30,854 (92.2)
Black patients	2262 (6.8)
Other patient subgroups	354 (1.1)
Ethnicity	
Not Spanish or Latino	32,970 (98.5)
Other	500 (1.5)
Smoking status	
Non-smoker	11,551 (34.5)
Smoker	12,079 (36.1)
Unknown or unspecified	9840 (29.4)
Marital status	
Married	4268 (12.8)
Single or never married	17,905 (53.5)
Widowed	3969 (11.9)
Divorced or separated	6139 (18.3)
Living with partner or unknown or not reported	1142 (3.6)
Appalachia status	
Non-Appalachia	23,126 (69.1)
Appalachia	10,344 (30.9)
Treatment group	
No or unknown or refused treatment	3366 (10.1)
Surgery	23,510 (70.2)
Chemotherapy and radiation	1191 (3.6)
Chemotherapy or radiation with surgery	2986 (8.9)
Other therapies	2417 (7.2)
Insurance type	
Private insurance	289 (0.9)
Government-related programs	21,563 (64.4)
Uninsured or self-pay	8293 (24.8)
Other programs	3325 (9.9)
Histology	
Adenocarcinoma NOS^b^	21,841 (65.3)
Papillary adenocarcinoma	2189 (6.5)
Tubulovillous adenocarcinoma	1126 (3.4)
Mucinous adenocarcinoma	2051 (6.1)
Other or unspecified	6263 (18.7)
Sentinel nodes	
Negative sentinel node	13,975 (41.8)
Sentinel nodes positive	8183 (24.4)
Unknown or other	11,312 (33.8)
SEER^c^ 2018 summary stage	
In situ, localized	4911 (14.7)
Regional, early spread	1645 (4.9)
Regional, more extensive spread	1282 (3.8)
Distant spread or metastasis	1563 (4.7)
Unknown or unspecified	24,069 (71.9)

a Analyses are based on a retrospective cohort study of patients with colon cancer using data from the Kentucky Cancer Registry collected between January 1, 2010, and December 31, 2022.

b NOS: not otherwise specified.

c SEER: Surveillance, Epidemiology, and End Results.

**Table 3.** Comparison of hazard ratios across racial, diagnosis age, and geographical subgroups using least absolute shrinkage and selection operator (LASSO) regression^a^.

Risk factor	Overall	White patients	Black patients	Early age^b^	Late age^c^	Non-Appalachia	Appalachia
Age (y)	1.035	1.035	1.033	NA^d^	NA	1.036	1.032
Treatment (reference=no or unknown or refused)							
Surgery (primary and regional)	0.309	0.304	0.321	0.297	0.280	0.327	0.289
Chemo and or radiation therapies	0.434	0.423	0.501	0.539	0.364	0.433	0.412
Surgery+(chemo/radiation)	0.264	0.260	0.286	0.269	0.202	0.277	0.246
Other therapies (immunotherapy, endoscopic, gene, etc)	0.622	0.610	0.68	0.653	0.430	0.680	0.558
Sex (reference=male)							
Female	0.865	0.859	0.897	0.929	0.853	0.855	0.901
Race (reference=White patients)							
Black patients	1.087	NA	NA	1.176	1.027	1.069	1.127
Other patient subgroups	0.546	NA	NA	0.449	0.548	0.554	0.381
Ethnicity (reference=not Spanish or Latino)							
Other	0.960	1.000	0.866	0.796	1.000	0.978	1.000
Marital status (reference=married)							
Single (never married)	0.779	0.776	0.842	0.813	0.845	0.774	0.807
Widowed	0.985	0.981	1.007	1.002	1.000	1.000	1.000
Divorced or separated	0.977	0.968	1.049	0.996	1.275	0.965	0.980
Living with partner or unknown or unreported	0.766	0.767	0.893	0.732	0.827	0.771	0.864
Smoking status (reference=non-smoker)							
Smoker (cigarettes, e-cigarette, cigar)	1.244	1.234	1.178	1.234	1.177	1.268	1.163
Smoker (unknown)	0.872	0.890	0.784	0.871	0.848	0.848	0.869
Cigarette pack years	1.002	1.002	1.000	1.004	1.001	1.001	1.003
Tumor grade (reference=localized)							
Regional by direct extension	1.149	1.003	1.126	1.273	1.156	1.031	1.000
Regional to lymph nodes	1.545	1.338	1.219	1.955	1.504	1.407	1.218
Regional by both direct extension and regional lymph nodes	1.419	1.224	1.221	1.843	1.360	1.332	1.158
Unknown or unstageable	1.259	1.088	1.000	1.289	1.244	1.076	1.179
Positive nodes (reference=all sentinel nodes examined are negative)							
Sentinel nodes are positive	1.538	1.555	1.668	1.838	1.502	1.559	1.586
Other or unknown	1.917	1.909	2.132	2.724	1.749	1.938	1.883
Tumor size	1.003	1.003	1.007	1.004	1.003	1.005	1.003
CS^e^ lymph nodes	1.001	1.001	1.000	1.000	1.001	1.001	1.001
Geographical region (reference=non-Appalachia)							
Appalachia	1.073	1.077	1.000	1.053	1.012	NA	NA
Histology (reference=adenocarcinoma NOS^f^)							
Papillary adenocarcinoma	0.585	0.586	0.617	0.416	0.612	0.599	0.599
Tubulovillous adenocarcinoma	0.268	0.281	0.217	0.123	0.347	0.280	0.248
Mucinous adenocarcinoma	1.058	1.051	1.051	1.054	1.056	1.076	1.000
Other or unspecified	0.791	0.811	0.695	0.592	0.852	0.825	0.793
Insurance (reference=private insurance)							
Government-related programs	0.985	1.000	1.000	0.953	1.091	1.000	0.981
Uninsured or self-pay	0.796	0.814	0.827	0.727	0.697	0.825	0.780
Other or unknown payers	1.227	1.278	1.036	1.211	1.385	1.304	1.181
SEER^g^ 2018 summary (reference=in situ, localized)							
Regional, early spread	1.581	1.548	1.190	1.698	1.585	1.595	1.364
Regional, more extensive spread	1.112	1.121	0.795	1.253	1.146	1.103	1.000
Distant spread or metastasis	1.325	1.298	1.170	1.868	1.284	1.213	1.411
Unknown or unspecified	1.974	1.949	1.621	3.297	1.797	1.976	1.832

a Analyses are based on a retrospective cohort study of patients with colon cancer using data from the Kentucky Cancer Registry collected between January 1, 2010, and December 31, 2022.

b Early age: early-onset of colon cancer (18-60 y).

c Late age: late-onset colon cancer (over 60 y).

d NA: not applicable.

e CS: clinical stage.

f NOS: not otherwise specified.

g SEER: Surveillance, Epidemiology, and End Results.

**Table 4.** Comparison of concordance index (C-index) for population subgroups for the fitted models^a^.

Subgroups	C-index for population subgroups models					
	Cox	AFT^b^	XGBoost^c^	LASSO^d^	Elastic net	RSF^e^
Overall	0.7605	0.7606	0.7007	0.7544	0.7569	0.8141
Appalachia	0.7624	0.7626	0.6971	0.7607	0.7614	0.8131
Non-Appalachia	0.7827	0.7819	0.7103	0.7581	0.7564	0.8101
Early diagnosis	0.7754	0.7755	0.7210	0.6852	0.7310	0.8158
Late diagnosis	0.7417	0.7420	0.7030	0.7369	0.7409	0.7832
White patients	0.7634	0.7633	0.7419	0.7598	0.7595	0.8161
Black patients	0.8037	0.8041	0.6889	0.7449	0.7469	0.7849

a Analyses are based on a retrospective cohort study of patients with colon cancer using data from the Kentucky Cancer Registry collected between January 1, 2010, and December 31, 2022.

b AFT: accelerated failure time.

c XGBoost: Extreme Gradient Boosting.

d LASSO: least absolute shrinkage and selection operator.

e RSF: random survival forest.

The RSF model demonstrated strong overall discriminatory performance, achieving a C-index of 0.8146, indicating a high level of agreement between predicted and observed survival outcomes. Model evaluation across key subgroups showed similar robust performance: patients diagnosed early (0.8175), patients diagnosed late (0.7841), Appalachia (0.8135), non-Appalachia (0.8126), White patients (0.8164), and Black patients (0.7881). The slightly higher C-index in the early diagnosis and White patient subgroups suggests marginally better discrimination in these populations, while the modestly lower score in the late diagnosis and Black patient subgroups still reflects acceptable performance. These results support the generalizability and reliability of the RSF model across diverse demographic and clinical strata. Overall, the model demonstrated stable predictive accuracy and effective risk stratification for colon cancer survival.

### Survival and Prevalence of Colon Cancer Death

**Figure 3.**
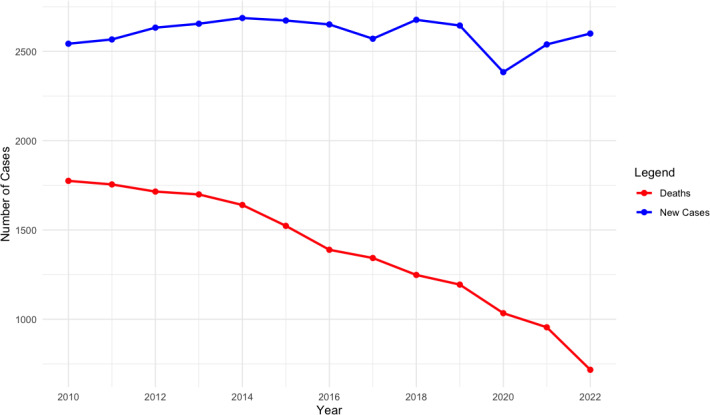
A comparison of new colorectal cancer (CRC) cases and deaths across the study period. Analyses are based on a retrospective cohort study of patients with colon cancer using data from the Kentucky Cancer Registry collected between January 1, 2010, and December 31, 2022.

**Figure 4.**
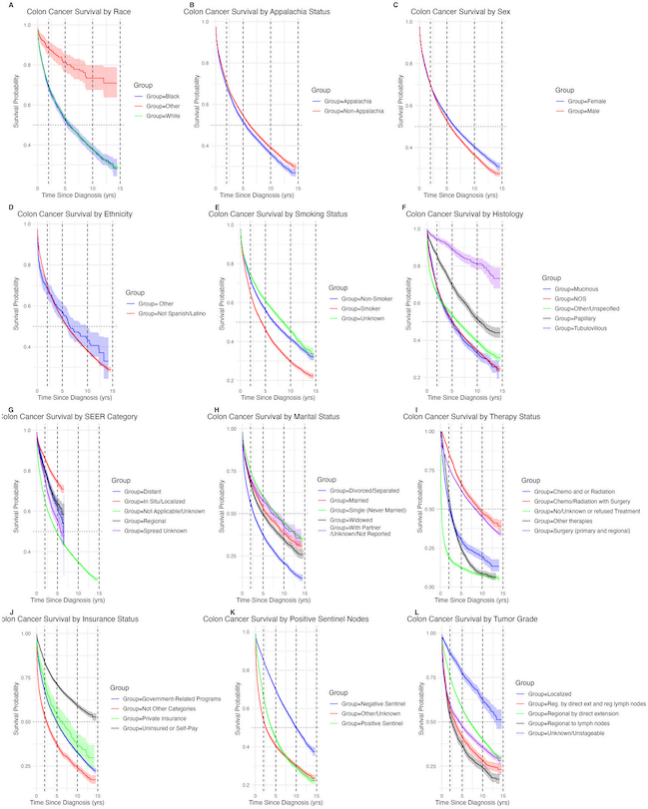
Kaplan-Meier colon cancer survival curves comparison colorectal cancer (CRC) cancer survival distribution by risk factors and time since diagnosis, with vertical dashes representing 2-, 5-, 10-, and 15-year survival probabilities. Analyses are based on a retrospective cohort study of patients with colon cancer using data from the Kentucky Cancer Registry collected between January 1, 2010, and December 31, 2022.

**Figure 5.**
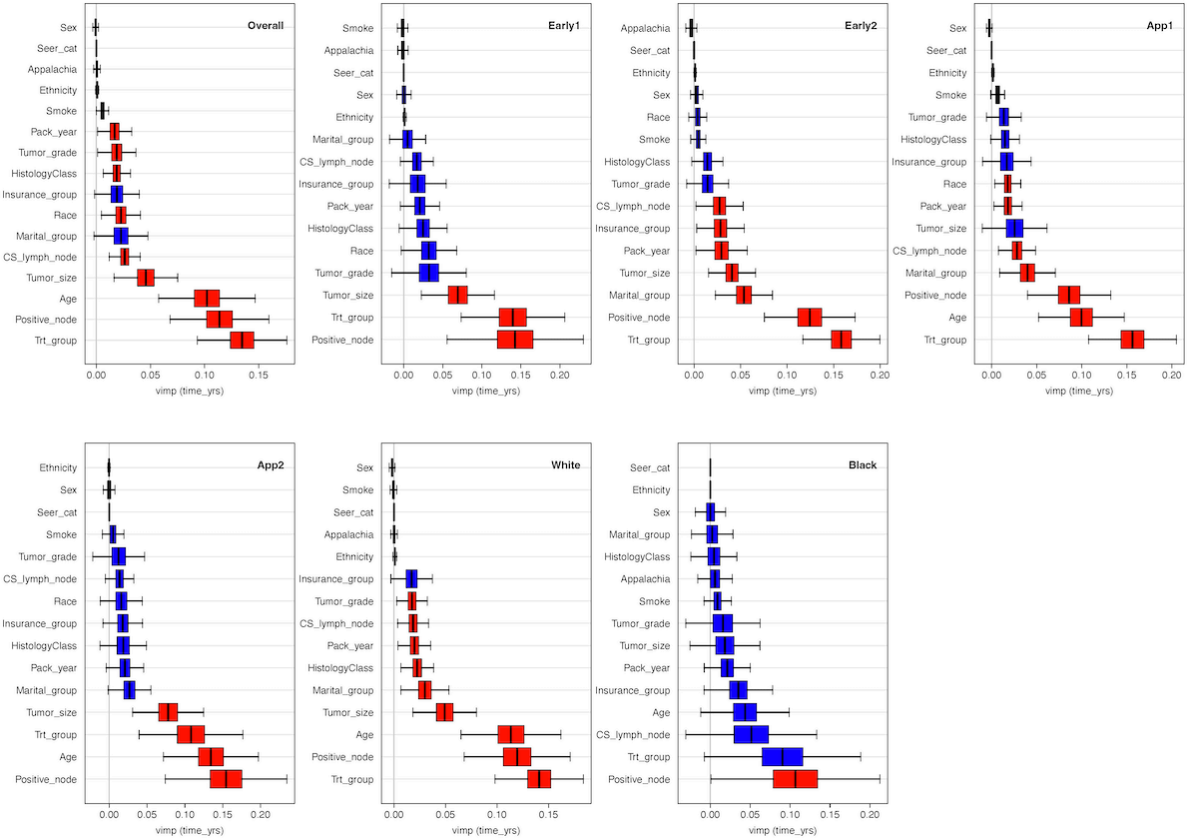
The overall ranking of risk factor importance and for each subgroup, as determined by the random survival forest model. Analyses are based on a retrospective cohort study of patients with colon cancer using data from the Kentucky Cancer Registry collected between January 1, 2010, and December 31, 2022. Overall is the overall model. “Overall” displays the significant risk factors in a "red" box plot and the nonsignificant ones in a "blue" box plot. “Early1” or “Early2” represent individuals diagnosed at early or late age, respectively. “App1” and “App2” correspond to results of individuals residing in non-Appalachia and Appalachia regions, respectively. “White” and “Black” show the results stratified by White and Black patient subgroups, respectively. Red and blue colored boxplots correspond to significant and nonsignificant risk factors, respectively.

Figure 3 shows the annual incidence and mortality of colon cancer, highlighting temporal trends including a slight dip in 2020 likely due to the COVID-19 pandemic disruption in screening and diagnosis. This decline may reflect delayed detection rather than a true reduction in CRC disease burden. Figure 4 shows Kaplan-Meier curves for CRC survival stratified by each risk factor, highlighting statistically significant differences between the distributions of at least 2 subgroups for each risk factor. Model accuracy for both the LASSO (*λ*=0.01) and elastic net (*λ*₁=0.02, *λ*₂=0) procedures was evaluated using leave-one cross-validation to minimize overfitting and enhance predictive performance. Elastic net results closely mirrored those of the LASSO and are omitted for brevity. Figure 5 illustrates the variable importance for the risk factors in the model. The plot highlights the order of importance, with the “red” boxplots indicating significant predictors of survival. These predictors are ranked based on their contribution to the model’s ability to accurately assess survival outcomes.

Table 4 shows the C-index values for the Cox, AFT, XGBoost, LASSO, elastic net, and RSF models across various subgroups. Among the ML models evaluated, RSF consistently achieved the highest C-index values, indicating superior discriminatory performance and a better overall fit to the survival data. This suggests that RSF is better able to capture complex, nonlinear relationships and interactions among the risk factors, which traditional models may overlook. While classical models like Cox and AFT performed reasonably well, their performance was generally outpaced by the more flexible ensemble-based RSF. These findings highlight the potential value of nonparametric ML approaches in improving predictive accuracy for survival outcomes.

Figure 1 displays the Brier scores by population subgroups for the Cox proportional hazards model. As shown, the Brier scores for the Cox model range between 0 and 0.25, indicating reasonably good predictive accuracy across the subgroups. Lower Brier scores reflect better performance in terms of both discrimination and calibration, suggesting that the Cox model fits the data relatively well. The consistency of low Brier scores across diverse subgroups further supports the model’s robustness among the classical methods. These results highlight the suitability of the Cox model for risk prediction in these population subgroups.

Figure 2 shows the evaluation of the predictive models from RSF for overall cohort and subgroups using the Brier score. The overall model showed consistent and stable performance at each point in time, while subgroup analyses indicated slightly higher Brier scores among Black patients, Appalachian residents, and those with late CRC diagnoses, suggesting modestly reduced calibration and predictive ability. However, the differences were not substantial, indicating no major bias in model performance across these groups.

### Model Results: Overall

In the Cox model, the most significant predictors of colon cancer survival, listed in order of importance (determined by *P* values), were positive nodes, treatment group, age, tumor size, CS lymph nodes, and tumor grade, with the remaining factors having no significant impact on survival (Table S2 in Multimedia Appendix 1, “Overall” column). The AFT model identified the same set of key predictors as the Cox model; sex was also significant in the AFT model, but the other predictors mentioned above were ranked in the same order for the AFT model (Table S1 in Multimedia Appendix 1, “Overall” column). However, in the RSF, the importance of risk factors differed slightly, with treatment group, positive nodes, age, clinical tumor size, CS lymph nodes, marital status, race, histology, insurance, cigarette pack years, tumor grade, smoking status, ethnicity, Appalachia, and sex following in descending order (Figure 5, overall panel). The effects of ethnicity, Appalachia, and sex were relatively small compared to those of other factors. The standardized OOB CRPS estimate was 0.155, with an OOB requested performance error of 0.254, indicating a very good fit of the model.

Table 3 presents the results for the LASSO model. For instance, the hazard ratio for overall age is 1.035, indicating that for each additional year of age, the hazard of death from colon cancer increased by 3.5%. Patients who underwent surgery (primary and regional) had a 69.1% lower risk of hazard compared to those who refused treatment or had no treatment, representing a significant reduction in risk. The most effective treatment regimen was surgery combined with either chemotherapy or radiation therapy, which showed the greatest benefit compared to all other treatment options. Female patients had a 13.5 lower hazard of colon cancer death compared to male patients (compare Figure 4 panel C). Black patients had an 8.7% higher risk of colon cancer death compared to White patients, even after adjusting for other risk factors in the model.

### Racial Subgroup

The significant risk factors for predicting colon cancer survival differ across racial groups, as identified by the AFT (Table S1 in Multimedia Appendix 1) and the Cox (Table S2 in Multimedia Appendix 1) models. Among White patients, the most important predictors are treatment, positive nodes, age, tumor size, tumor grade, CS lymph nodes, sex, and histology. For Black patients, however, the key predictors shift slightly, with positive nodes, smoking status, insurance, age, histology, CS lymph nodes, and cigarette pack years emerging as the primary factors. These differences highlight the potential impact of both genetic and lifestyle factors on survival outcomes in different racial groups.

In the RSF model for the White patients, the risk factors were ranked in the following order of importance: treatment group, positive nodes, age, tumor size, marital status, histology, cigarette pack years, CS lymph nodes, and tumor grade. The standardized OOB CRPS was 0.149, with an OOB requested performance error of 0.241. The rest of the risk factors had minimal impact on survival predictions. For Black patients, the key predictors included positive nodes, treatment group, CS lymph nodes, insurance, age, cigarette pack years, tumor size, and smoking. The standardized OOB CRPS was 0.184, with an OOB requested performance error of 0.327. All other risk factors contributed minimally to influence survival outcomes (Figure 5 panels White and Black patients). The above analysis was not repeated for the “Other” racial group due to limited data.

### Geographical Region

In both the AFT and Cox models, key risk factors for colon cancer survival vary between individuals living in the Appalachia region and those outside of it. For non-Appalachia residents, significant factors include treatment group, positive lymph nodes, age at diagnosis, tumor grade, marital status, and clinical tumor size (in the AFT and Cox models), with histology and CS lymph nodes also emerging as significant factors in the Cox model (Table S2 in Multimedia Appendix 1). In contrast, for Appalachia residents, survival is influenced by treatment group, age at diagnosis, positive lymph nodes, clinical tumor size, marital status, tumor grade, CS lymph node, and histology classification. The main differences between the 2 groups are the inclusion of CS lymph node and histology as significant factors for Appalachia residents, while clinical tumor size and smoking status are less relevant for non-Appalachia patients.

In the RSF model, the risk factors associated with colon cancer survival differ slightly between individuals living in Appalachia and those in non-Appalachia regions. For non-Appalachia residents (Figure 5, App1), the most influential factors, in order of importance, include treatment group, age at diagnosis, positive lymph nodes, marital status, CS lymph nodes, race, cigarette pack years, and smoking status. The impact of clinical tumor size was not quantified precisely but appeared to be high. Insurance, histology classification, and tumor grade also had high relevance for survival. The rest of the variables had a minimal impact. The standardized OOB CRPS was 0.151, and the OOB requested performance error was 0.244. In contrast, for patients in Appalachia (Figure 5, App2), the key significant risk factors were positive lymph nodes, age at diagnosis, treatment group, clinical tumor size, and smoking status. The standardized OOB CRPS was 0.191 and the OOB requested performance error of 0.353. The impact of cigarette pack years, histology, insurance, race, tumor grade, CS lymph nodes, and smoking status was not quantified precisely but appeared to be high. The primary differences lie in the order of importance, with treatment received and positive lymph nodes being important for both non-Appalachia and Appalachian residents, with factors such as marital status, insurance, and clinical tumor size being more prominent outside Appalachia.

### Early- Versus Late-Age Diagnosis

In both the Cox and AFT models, the risk factors for colon cancer survival differ slightly between early and late stages. For early diagnosis, the most significant risk factors across both models include positive nodes, treatment group, tumor grade, tumor size, histology, and race. In the Cox model, clinical tumor size is also an important factor, while the AFT model does not highlight this. For late diagnosis, both models identify treatment group, positive nodes, tumor size, marital status, and CS lymph node as key predictors. The Cox model ranked CS lymph node and tumor size higher than marital status, while the AFT model emphasizes CS lymph nodes and marital status with different weights (Tables S1 and S2 in Multimedia Appendix 1).

In the RSF model, the key risk factors for patients diagnosed at early age with colon cancer include positive nodes, treatment group, and tumor size. Other high-priority factors are tumor grade, race, histology, cigarette pack years, insurance, CS lymph nodes, and marital status, while the remaining factors have a minimal impact on survival (Figure 5, panel Early1). For patients diagnosed at an older age, the key risk factors shift slightly to include treatment group, positive nodes, marital status, clinical tumor size, insurance, cigarette pack years, CS lymph nodes, and histology. Tumor grade is also a high-priority factor. Race, smoking status, sex, ethnicity, and Appalachia status contribute minimally to survival outcomes (Figure 5, panel Early2).

## Discussion

### Key Findings

The main findings of this study highlight that several individual and contextual risk factors significantly influence colon cancer survival. Across almost all models, positive sentinel lymph node, age at diagnosis, treatment received, clinical tumor size, tumor grade, smoking, geographic region, and marital status consistently emerged as dominant predictors. The highest risk of death (Table 3) was observed among individuals who received no treatment or refused treatment, compared to those who underwent any other form of treatment (primary and regional). Social determinants of health such as insurance (which serves as a proxy for poverty), Appalachia region, marital status, and modifiable risk factors such as smoking significantly impact survival. Not having insurance, smoking, living in the Appalachia region, and being married are associated with higher risk of dying. The comparison of classical and ML models demonstrated that ML approaches, particularly RSF and LASSO, offered improved predictive accuracy and variable importance insights compared to traditional methods. Consistent with prior studies, these results affirm that men have a higher risk of colon cancer death than women [56-58].

Overall, smokers had a higher hazard of mortality compared to non-smokers, a finding consistent with the results reported by Tsao et al [59]. Additionally, Appalachian residents had higher hazard than non-Appalachians (Table 3). In non-Appalachian residents, the hazard of death was higher for those with distant metastasis compared to those with in situ localized cancer diagnosis. In contrast, Appalachian residents had a higher hazard of death with distant metastasis, suggesting an interaction between geography and cancer spread, which may be compared with the findings of Wang et al [60].

### Model Comparisons

When comparing significant risk factors across racial groups in the Cox model, it was evident that while the type of treatment received was a significant predictor of survival for the White patients, it did not appear to be a useful predictor for the Black patients (Table S2 in Multimedia Appendix 1). This disparity may highlight the possibility that Black patients often seek treatment later in the disease course, potentially reducing the effectiveness of the treatment they receive. However, modifiable risk factors such as smoking, cigarette pack years, and insurance status were found to impact survival among Black patients compared to White patients. This suggests that addressing these factors could help narrow the gap in colon cancer survival between White and Black patients.

In the RSF model, differences emerged in how treatment and socioeconomic factors (like insurance and smoking history) influence survival outcomes across racial groups. Both groups shared predictors like positive nodes, clinical tumor size, and tumor grade, but White patients’ survival was more impacted by treatment received than positive node, while Black patients’ survival was more impacted by positive node than treatment received. Other important risk factors affecting survival include smoking and insurance, which showed differences compared with White patients. This contrast suggests that while biological factors (such as tumor characteristics) are universally relevant, socioeconomic and access disparities exacerbate survival gaps between racial groups. Overall, positive lymph nodes, treatment, age at diagnosis, CS lymph nodes, tumor size, tumor grade, and histology were important risk factors in both Appalachian and non-Appalachian areas of Kentucky. CS lymph nodes, however, were more influential within the non-Appalachian population compared to patients in Appalachia.

For the Cox model, while positive nodes, treatment group, and histology were consistently significant for both early and late diagnosis, tumor characteristics and social risk factors like marital status varied between the 2 groups (early vs late diagnosis). These findings suggest that we need to integrate critical risk factors into individualized prognostic models.

### Strengths

The key strength of this study is its comprehensive comparison of both classical and ML survival models and using large, population-based cancer registry data. By evaluating multiple modeling approaches including classical methods like Cox proportional hazards, AFT models, RSF, LASSO, and elastic net, our study offers valuable insights into the strengths and limitations of each method in predicting key risk factors associated with colon cancer survival. This side-by-side evaluation (Tables3  4, Tables S1 and S2 in Multimedia Appendix 1, and Figures 1-5) enhances our understanding of how different algorithms handle clinical and demographic risk factors, ultimately contributing to more accurate and interpretable survival predictions. The inclusion of subgroup analyses further strengthens the findings by highlighting how predictive performance and risk factors vary across different population subgroups.

### Limitations and Future Directions

First, a major limitation of this study is the lack of access to detailed, case-level data, which prevented us from analyzing several important modifiable risk factors such as alcohol consumption, obesity, familial associations or family history of CRC, inflammatory bowel disease, and dietary intake (including consumption of processed and red meats). These factors are well-established contributors to CRC risk and may also influence survival outcomes. The absence of these variables could lead to residual confounding and limits our ability to fully characterize the risk profile of patients. Future studies incorporating more granular lifestyle and clinical data would provide a more comprehensive understanding of survival determinants and allow for the evaluation of modifiable behavioral risk factors.

### Clinical Implication

The findings from our survival analysis models (Kaplan-Meier curve, Cox, AFT, XGBoost, RSF, LASSO, and elastic net) provide several actionable insights for both clinical practice and public health. Across all models, consistent predictors such as positive lymph node involvement, tumor size, tumor grade, and age were associated with both early and late diagnoses of CRC. Clinically, this supports the development of risk-stratified care pathways, where patients presenting with these characteristics can be prioritized for more intensive surveillance and earlier intervention. For example, individuals with larger or high-grade tumors may benefit from more frequent imaging or expedited treatment planning. Incorporating these factors into clinical decision-making tools could help optimize resource allocation and improve outcomes through early detection and treatment.

Beyond clinical implications, the models also highlight social and structural determinants of health that warrant attention. Race and insurance status, which were significant in the AFT and RSF models, suggest potential disparities in access to timely diagnosis. These findings can inform public health strategies such as targeted screening and outreach programs in underserved communities. The significance of marital status, particularly in late diagnosis, points to the role of social support in health care engagement, indicating a need to connect socially isolated individuals with navigators or support services. Additionally, the inclusion of cigarette pack-years and CS lymph node involvement as key factors further supports integrating lifestyle and clinical history into risk prediction frameworks, enhancing early identification of high-risk patients in both clinical and community settings. Together, these findings support the creation of risk-adapted screening, surveillance, and treatment pathways, as well as inform broader policy interventions aimed at improving access and reducing disparities in CRC outcomes.

### Colon Cancer Survival and Prognosis

Different subgroups may have varying risk profiles influencing colon cancer survival and prognosis, highlighting the limitations of a one-size-fits-all approach. Understanding subgroup-specific risk factors enables tailored interventions, screening, and treatment strategies for diverse populations. By leveraging results from classical and ML survival models including Cox, AFT, RSF, LASSO, and elastic net methods, clinicians and researchers can identify the most relevant predictors of patients’ survival outcome and develop individualized risk assessments. For example, the AFT model indicates that among early-onset patients, smokers experience death approximately 10% sooner than never-smokers, whereas in late-onset patients, this difference increases to 13%. Such insights highlight modifiable risk factors like smoking and suggest prioritization for targeted intervention in the higher-risk subgroups.

Colon cancer survival outcomes are shaped by both modifiable risk factors, such as smoking, insurance status, and geographic disparities, and non-modifiable factors, including age, genetics, and comorbidities. Early detection through screening remains the most effective strategy [61,62], as it significantly improves survival by enabling timely and effective treatment. Integrating these findings across different subgroups supports more precise, personalized treatment and preventive strategies, ultimately reducing the burden of colon cancer at both individual and population levels.

### Conclusion

This study evaluated multiple survival analysis models, including the Cox proportional hazards model (Cox model), the AFT model, XGBoost, RSF, the LASSO regression, and elastic net. Each of these models has distinct strengths and assumptions, making them suitable for different aspects of our research questions. Both LASSO and elastic net are powerful regularization techniques that helped improve the model generalization, interpretability, and predictive accuracy.

## Supplementary material

10.2196/72665 Multimedia Appendix 1 Parameter estimates from both the accelerated time model and Cox model, shown for the overall cohort and for each subgroup.

## Abbreviations

AFT: accelerated failure time
AJCC: American Joint Committee on Cancer
C-index: concordance index
CRC: colorectal cancer
CRPS: Continuous Ranked Probability Score
CS: clinical stage
KCR: Kentucky Cancer Registry
LASSO: least absolute shrinkage and selection operator
ML: machine learning
OOB: out-of-bag
RSF: random survival forest
SEER: Surveillance, Epidemiology, and End Results
STROBE: Strengthening the Reporting of Observational Studies in Epidemiology
TNM: tumor, nodes, and metastasis
TRIPOD+AI: Transparent Reporting of a Multivariable Prediction Model for Individual Prognosis or Diagnosis Using Artificial Intelligence
XGBoost: Extreme Gradient Boosting
