# Supplementary material for: Comparison of Machine Learning Models for Colon Cancer Survival: Predictive Modeling Approach
Source: JMIR Cancer. 2025 Nov 26;11:e72665. doi: 10.2196/72665 (PMC12655889; doi:10.2196/72665)
Supplement: Multimedia Appendix 1 [file cancer-v11-e72665-s001.docx]

**Supplementary Tables**

Table 1: Log parameter estimates from the accelerated failure time model for the overall population and across the different subgroups.

|  | **Overall** | | **White Patients** | | **Black Patients** | | **Diagnosis**  **Early Late** | | | | **Appalachia**  **No Yes** | | | |
| --- | --- | --- | --- | --- | --- | --- | --- | --- | --- | --- | --- | --- | --- | --- |
| **Risk factor** | **Est**  **(SE)** | **P-Val** | **Est**  **(SE)** | **P-Val** | **Est**  **(SE)** | **P-Val** | **Est**  **(SE)** | **P-Val** | **Est**  **(SE)** | **P-Val** | **Est**  **(SE)** | **P-Val** | **Est**  **(SE)** | **P-Val** |
| Intercept | 3.287 (0.8983) | 2.53E-04 | 3.172  (0.9129) | 5.12E-04 | 5.413 (2.2062) | 0.0141 | 1.378 (1.2408) | 2.67E-01 | 1.821 (1.2081) | 1.32E-01 | 3.094 (1.2235) | 1.15E-02 | 4.514 (1.393) | 1.19E-03 |
| Age | -0.026  (0.0047) | 2.14E-08 | -0.026 (0.0049) | 1.83E-07 | -0.069 (0.0233) | 0.0033 | NA | NA | NA | NA | -0.023 (0.0053) | 1.42E-05 | -0.050 (0.0104) | 1.57E-06 |
| **Treatment (Ref = No/Unknown or Refused)** | | | | | | | | | | | | | | |
| Surgery (primary and regional) | 1.540 (0.1901) | 5.51E-16 | 1.613 (0.2065) | 5.75E-15 | -0.146 (0.5923) | 0.8053 | 1.560 (0.3981) | 8.89E-05 | 1.637 (0.2316) | 1.58E-12 | 1.556 (0.2254) | 5.15E-12 | 1.858 (0.384) | 1.31E-06 |
| Chemo and or radiation therapies | 1.809 (0.2167) | 6.77E-17 | 1.934 (0.2256) | 9.97E-18 | 0.377 (0.7359) | 0.6084 | 1.507 (0.4402) | 6.19E-04 | 2.088 (0.2708) | 1.25E-14 | 1.917 (0.2703) | 1.30E-12 | 2.157 (0.4033) | 8.93E-08 |
| Surgery + (chemo/radiation) | 1.617 (0.2217) | 3.10E-13 | 1.731 (0.2352) | 1.88E-13 | -0.424 (0.723) | 0.5577 | 1.827 (0.4437) | 3.84E-05 | 1.737 (0.2636) | 4.49E-11 | 1.600 (0.2708) | 3.43E-09 | 2.065 (0.411) | 5.06E-07 |
| Other therapies (immunotherapy, endoscopic, gene, etc.) | 0.574 (0.2058) | 5.31E-03 | 0.669 (0.2211) | 2.49E-03 | -0.451 (0.806) | 0.5754 | 0.706 (0.3986) | 7.65E-02 | 0.763 (0.2585) | 3.17E-03 | 0.331 (0.2391) | 1.66E-01 | 1.574 (0.4171) | 1.61E-04 |
| **Sex** | | | | | | | | | | | | | | |
| Female | 0.210 (0.1016) | 3.88E-02 | 0.187 (0.1058) | 7.81E-02 | 0.754 (0.4653) | 0.1052 | 0.343 (0.1881) | 6.84E-02 | 0.120 (0.1274) | 3.47E-01 | 0.225 (0.1172) | 5.51E-02 | -0.2113 (0.2083) | 3.10E-01 |
| **Race (Ref = White Patients)** | | | | | | | | | | | | | | |
| Black Patients | 0.134 (0.181) | 4.60E-01 | NA | NA | NA | NA | 0.145 (0.3156) | 6.46E-01 | 0.077 (0.2345) | 7.42E-01 | 0.166 (0.1906) | 3.83E-01 | 0.011 (0.5992) | 9.85E-01 |
| Other Patient Subgroups | -0.305  (0.4872) | 5.27E-01 | NA | NA | NA | NA | -1.233 (0.6254) | 4.86E-02 | 0.278 (0.7852) | 7.24E-01 | -0.184 (0.4815) | 7.02E-01 | NA | NA |
| **Ethnicity (Ref = Not Spanish or Latino)** | | | | | | | | | | | | | | |
| Other | 0.252 (0.6189) | 6.84E-01 | 0.237 (0.6175) | 7.01E-01 | NA | NA | 1.003 (1.0564) | 3.43E-01 | -0.133 (0.7792) | 8.64E-01 | 0.575 (0.7559) | 4.47E-01 | -0.066 (1.0092) | 9.48E-01 |
| **Marital Status (Ref = Married)** | | | | | | | | | | | | | | |
| Single (never married) | 0.224  (0.1431) | 1.17E-01 | 0.207 (0.1541) | 1.80E-01 | -0.711 (0.4865) | 0.144 | 0.225 (0.212) | 2.88E-01 | 0.079 (0.208) | 7.05E-01 | 0.149 (0.1618) | 3.59E-01 | 0.883 (0.3012) | 3.37E-03 |
| Widowed | -0.086  (0.1772) | 6.26E-01 | -0.140 (0.1869) | 4.52E-01 | 0.281 (0.7848) | 0.7207 | -0.163 (0.2623) | 5.35E-01 | -0.209 (0.2526) | 4.08E-01 | 0.004 (0.2102) | 9.87E-01 | 0.200 (0.3321) | 5.46E-01 |
| Divorced/separated | -0.249 (0.1702) | 1.44E-01 | -0.311 (0.1828) | 8.94E-02 | 0.446 (0.598) | 0.4562 | -0.359 (0.4708) | 4.46E-01 | -0.497 (0.2153) | 2.11E-02 | -0.426 (0.1934) | 2.75E-02 | 0.669 (0.3645) | 6.66E-02 |
| Living with partner/unknown/unreported | -0.495 (0.3605) | 1.70E-01 | -0.533 (0.3826) | 1.64E-01 | -0.794 (0.9905) | 0.4226 | -0.687 (0.7633) | 3.68E-01 | -0.575 (0.4404) | 1.92E-01 | -0.718 (0.4241) | 9.07E-02 | 0.510 (0.69) | 4.60E-01 |
| **Smoking Status (Ref = Never smoker)** | | | | | | | | | | | | | | |
| Smoker (cigarettes, e-cigarette, cigar) | -0.121  (0.1266) | 3.40E-01 | -0.152 (0.1321) | 2.51E-01 | 1.366 (0.4502) | 0.0024 | -0.099 (0.2191) | 6.52E-01 | -0.134 (0.1601) | 4.02E-01 | -0.011 (0.1459) | 9.43E-01 | -0.503 (0.2587) | 5.19E-02 |
| Smoker (unknown) | -1.796  (1.0746) | 9.46E-02 | NA | NA | -1.638 (0.9538) | 0.0859 | NA | NA | -1.618 (1.1183) | 1.48E-01 | -1.835 (1.0687) | 8.61E-02 | NA | NA |
|  | | | | | | | | | | | | | | |
| Cigarette Pack Years | -0.001  (0.0021) | 5.70E-01 | -7e-04 (0.0022) | 7.42E-01 | -0.019 (0.0111) | 0.0866 | -0.004 (0.0044) | 3.68E-01 | 1E-04 (0.0026) | 9.63E-01 | -0.002 (0.0025) | 5.43E-01 | -4E-04 (0.0043) | 9.24E-01 |
| **Tumor Grade (Ref= Localized)** | | | | | | | | | | | | | | |
| Regional by direct extension | -0.657 (0.2894) | 2.32E-02 | -0.585 (0.3053) | 5.56E-02 | -0.143 (0.8674) | 0.8695 | -0.690 (0.4842) | 1.54E-01 | -0.538 (0.3613) | 1.37E-01 | -0.657 (0.3642) | 7.12E-02 | -1.026 (0.4901) | 3.64E-02 |
| Regional to lymph nodes | -1.031 (0.3201) | 1.28E-03 | -1.011 (0.3359) | 2.61E-03 | 0.238 (1.2723) | 0.8517 | -1.479 (0.5522) | 7.81E-03 | -0.848 (0.3981) | 3.31E-02 | -1.179 (0.4011) | 3.28E-03 | -1.148 (0.5286) | 2.99E-02 |
| Regional by both direct extension and regional lymph nodes | -0.853 (0.3184) | 7.39E-03 | -0.810 (0.3367) | 1.61E-02 | -1.046 (0.9808) | 0.2863 | -1.704 (0.5635) | 2.49E-03 | -0.505 (0.3923) | 1.98E-01 | -0.879 (0.3998) | 2.79E-02 | -1.084 (0.5347) | 4.26E-02 |
| Unknown/Unstageable | -0.482 (0.3054) | 1.15E-01 | -0.330 (0.3275) | 3.14E-01 | -0.267 (0.9357) | 0.7752 | -1.137 (0.5212) | 2.91E-02 | -0.184 (0.3879) | 6.35E-01 | -0.354 (0.389) | 3.63E-01 | -0.983 (0.5039) | 5.11E-02 |
| **Positive Nodes (Ref = All sentinel nodes examined are negative)** | | | | | | | | | | | | | | |
| Sentinel nodes are positive | -0.298  (0.1586) | 5.99E-02 | -0.248 (0.1668) | 1.37E-01 | -0.346 (0.5013) | 0.4901 | -0.290 (0.2746) | 2.91E-01 | -0.202 (0.2067) | 3.29E-01 | -0.243 (0.1956) | 2.15E-01 | -0.232 (0.2752) | 3.98E-01 |
| Other/Unknown | -1.289 (0.1434) | 2.51E-19 | -1.316 (0.1492) | 1.16E-18 | -2.011 (0.5881) | 0.0006 | -1.379 (0.2587) | 9.77E-08 | -1.281 (0.181) | 1.49E-12 | -1.178 (0.1692) | 3.25E-12 | -1.517 (0.2752) | 3.56E-08 |
|  | | | | | | | | | | | | | | |
| **Clinical Tumor Size** | -0.003  (7e-04) | 1.86E-06 | -0.003  (7e-04) | 7.85E-06 | -0.001 (0.0067) | 0.9447 | -0.003 (9E-04) | 1.94E-03 | -0.005 (0.0019) | 5.52E-03 | -0.005 (0.0021) | 2.53E-02 | -0.003 (8E-04) | 1.15E-03 |
| **Geographical Region (Ref = non-Appalachia)** | | | | | | | | | | | | | | |
| Appalachia | 0.050 (0.1101) | 6.49E-01 | 0.047 (0.1121) | 6.76E-01 | -0.290 (0.5181) | 0.576 | 0.164 (0.1971) | 4.05E-01 | 0.095 (0.1358) | 4.86E-01 | NA | NA | NA | NA |
| **Histology (Ref =** Adenocarcinoma NOS**)** | | | | | | | | | | | | | | |
| Papillary Adenocarcinoma | 0.102 (0.2147) | 6.34E-01 | 0.037 (0.2184) | 8.67E-01 | 0.605 (0.9711) | 0.5331 | 0.585 (0.4108) | 1.54E-01 | -0.111 (0.2588) | 6.69E-01 | -0.012 (0.2486) | 9.61E-01 | -9E-04 (0.416) | 9.98E-01 |
| Tubulovillous Adenocarcinoma | 0.676  (0.4473) | 1.31E-01 | 0.848 (0.4796) | 7.71E-02 | 1.217 (1.298) | 0.3484 | 1.660 (0.7681) | 3.07E-02 | 0.625 (0.5585) | 2.63E-01 | 0.101 (0.5395) | 8.51E-01 | 1.870 (0.7959) | 1.88E-02 |
| Mucinous Adenocarcinoma | -0.342 (0.1775) | 5.42E-02 | -0.363 (0.1801) | 4.40E-02 | -0.785 (0.9421) | 0.4046 | 0.293 (0.3596) | 4.15E-01 | -0.433 (0.2128) | 4.18E-02 | -0.466 (0.1916) | 1.50E-02 | 0.271 (0.4639) | 5.60E-01 |
| Other/Unspecified | 0.006 (0.1378) | 9.67E-01 | -0.045 (0.1419) | 7.51E-01 | 0.975 (0.4436) | 0.028 | 0.418 (0.2781) | 1.33E-01 | -0.139 (0.1666) | 4.05E-01 | -0.040 (0.1719) | 8.16E-01 | -0.042 (0.2238) | 8.50E-01 |
|  | | | | | | | | | | | | | | |
| **CS Lymph Nodes** | -0.001 (5e-04) | 2.47E-03 | -0.001  (5e-04) | 8.18E-03 | -0.004 (0.0014) | 0.008 | -3E-04 (7E-04) | 6.88E-01 | -0.002 (7E-04) | 7.78E-04 | -0.001 (6E-04) | 5.01E-02 | -0.002 (7E-04) | 1.35E-02 |
| **Insurance (Ref = Private Insurance)** | | | | | | | | | | | | | | |
| Government-Related Programs | 0.393 (0.7539) | 6.03E-01 | 0.340 (0.7524) | 6.52E-01 | 1.870 (0.6483) | 0.0039 | 0.749 (1.0321) | 4.68E-01 | 0.010  (1.1026) | 9.93E-01 | 0.441 (1.0636) | 6.78E-01 | 0.398 (1.0225) | 6.97E-01 |
| Uninsured or Self-Pay | 0.910 (0.7581) | 2.30E-01 | 0.885 (0.7570) | 2.43E-01 | 1.790 (0.6881) | 0.0093 | 1.115 (1.0277) | 2.78E-01 | 0.941 (1.1216) | 4.01E-01 | 0.953 (1.0606) | 3.69E-01 | 0.814 (1.0507) | 4.39E-01 |
| Other/Unknown Payers | 0.051 (0.7686) | 9.47E-01 | 0.082 (0.7695) | 9.15E-01 | NA | NA | 0.475 (1.0817) | 6.60E-01 | -0.318 (1.1142) | 7.75E-01 | -0.171 (1.0796) | 8.74E-01 | 0.454 (1.0561) | 6.68E-01 |
| Log(scale) | 0.048 (0.0375) | 2.01E-01 | 0.044 (0.0392) | 2.60E-01 | -0.292 (0.1341) | 0.0297 | -0.012 (0.0687) | 8.57E-01 | 0.074 (0.0445) | 9.71E-02 | 0.037 (0.0437) | 3.96E-01 | -0.034 (0.0722) | 6.41E-01 |

Footnote: Analyses are based on a retrospective cohort study of colon cancer patients using data from the Kentucky Cancer Registry collected between January 1, 2010, and December 31, 2022_._ Est (SE): Estimated log parameter from the accelerated failure time model (standard error), NOS: Not Otherwise Specified, P-val: Adjusted p-value. For a categorical risk factor, a negative estimate means accelerated deterioration of survival probability for people in the corresponding category compared to the reference. Thus, negative estimates identify unfavorable categories.

Table 2: Log hazard ratio estimates from Cox model for the overall population and across the subgroups.

|  | **Overall** | | **White Patients** | | **Black Patients** | | **Early Diagnosis** | | **Late Diagnosis** | | **Non-Appalachia** | | **Appalachia** | |
| --- | --- | --- | --- | --- | --- | --- | --- | --- | --- | --- | --- | --- | --- | --- |
| **Risk factors** | **Est**  **(SE)** | **P-Val** | **Est**  **(SE)** | **P-Val** | **Est**  **(SE)** | **P-Val** | **Est**  **(SE)** | **P-Val** | **Est**  **(SE)** | **P-Val** | **Est**  **(SE)** | **P-Val** | **Est (SE)** | **P-Val** |
| Age | 0.026  (0.0045) | 1.2268E-08 | 0.025  (0.0047) | 1.1807E-07 | 0.088  (0.0316) | 0.005  26566 | NA | NA | NA | NA | 0.023  (0.0052) | 1.1525E-05 | 0.053  (0.0107) | 8.8763E-07 |
| **Treatment (Ref = No/Unknown or Refused)** | | | | | | | | | | | | | | |
| Surgery (primary and regional) | -1.476  (0.1839) | 1.0384E-15 | -1.553  (0.2016) | 1.3559E-14 | 0.010  (0.8018) | 0.98993533 | -1.640  (0.405) | 5.1105E-05 | -1.526  (0.2196) | 3.6374E-12 | -1.502  (0.2207) | 1.0095E-11 | -1.965  (0.4145) | 2.1392E-06 |
| Chemo and or radiation therapies | -1.748  (0.2111) | 1.2293E-16 | -1.875  (0.222) | 3.0569E-17 | -0.782  (0.9953) | 0.43178175 | -1.603  (0.4543) | 0.00041565 | -1.962  (0.2581) | 2.912E-14 | -1.857  (0.2661) | 2.9558E-12 | -2.295  (0.4405) | 1.8897E-07 |
| Surgery + (chemo/radiation) | -1.541  (0.2132) | 4.9807E-13 | -1.659  (0.2282) | 3.6312E-13 | 0.395  (0.9838) | 0.68776855 | -1.925  (0.4497) | 1.8734E-05 | -1.598  (0.2482) | 1.1951E-10 | -1.537  (0.2634) | 5.3718E-09 | -2.183  (0.444) | 8.775E-07 |
| Other therapies (immunotherapy, endoscopic, gene, etc.) | -0.537  (0.1979) | 0.00660985 | -0.63  (0.2146) | 0.00330889 | 0.560  (1.0734) | 0.60209479 | -0.795  (0.4102) | 0.05244838 | -0.676  (0.2419) | 0.00518855 | -0.305  (0.2317) | 0.188536 | -1.658  (0.4496) | 0.00022735 |
| **Sex** | | | | | | | | | | | | | | |
| Female | -0.201  (0.0968) | 0.03767692 | -0.180  (0.1013) | 0.07553708 | -0.861  (0.627) | 0.16964131 | -0.341  (0.1896) | 0.07220556 | -0.112  (0.1184) | 0.3444017 | -0.214 (0.1129) | 0.05757586 | 0.223(0.2163) | 0.3027006 |
| **Race (Ref = White Patients)** | | | | | | | | | | | | | | |
| Black Patients | -0.127  (0.1728) | 0.4611241 | NA | NA | NA | NA | -0.177  (0.3215) | 0.5828042 | -0.063  (0.2182) | 0.7719591 | -0.161  (0.1842) | 0.3812196 | -0.008  (0.6294) | 0.9904609 |
| Other Patient Subgroups | 0.304  (0.4602) | 0.5089718 | NA | NA | NA | NA | 1.211  (0.6331) | 0.05566379 | -0.225  (0.7296) | 0.7574527 | 0.19  (0.4641) | 0.6815312 | NA | NA |
| **Ethnicity (Ref = Not Spanish or Latino)** | | | | | | | | | | | | | | |
| Other | -0.236  (0.5899) | 0.6889308 | -0.226  (0.5909) | 0.7017664 | NA | NA | -0.935  (1.0675) | 0.3810444 | 0.165  (0.723) | 0.8199719 | -0.559  (0.728) | 0.4428463 | 0.126  (1.0459) | 0.9042958 |
| **Marital Status (Ref = Married)** | | | | | | | | | | | | | | |
| Single (never married) | -0.218  (0.1366) | 0.1103072 | -0.201  (0.1477) | 0.1739945 | 0.878  (0.6634) | 0.18566773 | -0.221  (0.2154) | 0.3042459 | -0.09  (0.1932) | 0.6424557 | -0.150  (0.1562) | 0.3366859 | -0.931  (0.3134) | 0.00297472 |
| Widowed | 0.089  (0.169) | 0.5983071 | 0.145  (0.179) | 0.4176102 | -0.450  (1.026) | 0.66065474 | 0.182  (0.2651) | 0.4930478 | 0.189  (0.2346) | 0.4192643 | -0.001  (0.2027) | 0.9946225 | -0.193  (0.3445) | 0.5758661 |
| Divorced/separated | 0.233  (0.1622) | 0.1517788 | 0.297  (0.1749) | 0.08896344 | -0.840  (0.8373) | 0.31583105 | 0.297  (0.4795) | 0.5349386 | 0.453  (0.1993) | 0.02313812 | 0.402  (0.186) | 0.03085635 | -0.698  (0.3768) | 0.06399579 |
| Living with partner/unknown/unreported | 0.481  (0.344) | 0.1618684 | 0.524  (0.3665) | 0.1531652 | 1.067  (1.3668) | 0.43502278 | 0.681  (0.7692) | 0.3760825 | 0.526  (0.4095) | 0.1990397 | 0.703  (0.4092) | 0.08562187 | -0.593  (0.7168) | 0.4077564 |
| **Smoking Status (Ref = Never smoker)** | | | | | | | | | | | | | | |
| Smoker (cigarettes, e-cigarette, cigar) | 0.113  (0.1208) | 0.3507089 | 0.144  (0.1266) | 0.2567869 | -1.788  (0.6389) | 0.00513185 | 0.089  (0.2228) | 0.6882067 | 0.123  (0.1488) | 0.4095923 | 0.008  (0.1406) | 0.9566905 | 0.510  (0.2678) | 0.05672918 |
| Smoker (unknown) | 1.801  (1.0252) | 0.07890017 | NA | NA | 2.418  (1.3212) | 0.06728618 | NA | NA | 1.623  (1.0399) | 0.1185564 | 1.849  (1.0312) | 0.07288724 | NA | NA |
|  | | | | | | | | | | | | | | |
| Cigarette Pack Years | 0.001  (0.002) | 0.5562048 | 0.001  (0.0021) | 0.7320949 | 0.025  (0.015) | 0.0924417 | 0.004  (0.0044) | 0.3503019 | 0.000  (0.0024) | 0.9574962 | 0.001  (0.0024) | 0.5296856 | 0.001  (0.0045) | 0.8923298 |
| **Tumor Grade (Ref= Localized)** | | | | | | | | | | | | | | |
| Regional by direct extension | 0.627  (0.2756) | 0.02290213 | 0.564  (0.292) | 0.05329258 | 0.049  (1.1609) | 0.9661136 | 0.666  (0.4894) | 0.1735266 | 0.505  (0.3356) | 0.1326833 | 0.627  (0.3501) | 0.07317424 | 1.082  (0.5069) | 0.032765 |
| Regional to lymph nodes | 0.981  (0.3044) | 0.00126432 | 0.971  (0.3209) | 0.00249059 | -0.552  (1.7036) | 0.74591625 | 1.450  (0.5561) | 0.00910546 | 0.785  (0.3698) | 0.03385527 | 1.127  (0.3852) | 0.00344817 | 1.218  (0.5496) | 0.02667463 |
| Regional by both direct extension and regional lymph nodes | 0.813  (0.3033) | 0.00732828 | 0.778  (0.322) | 0.01572659 | 1.303  (1.2909) | 0.31277503 | 1.649  (0.5698) | 0.00381321 | 0.473  (0.3649) | 0.1950488 | 0.834(0.3842) | 0.02994596 | 1.153(0.5549) | 0.037649 |
| Unknown/Unstageable | 0.460  (0.2906) | 0.1138472 | 0.315  (0.313) | 0.3146858 | 0.345  (1.2546) | 0.78335678 | 1.132  (0.5254) | 0.03114618 | 0.176  (0.3602) | 0.6256746 | 0.341  (0.3736) | 0.3608848 | 1.067  (0.5217) | 0.04086074 |
| **Positive Nodes (Ref = All sentinel nodes examined are negative)** | | | | | | | | | | | | | | |
| Sentinel nodes are positive | 0.280  (0.151) | 0.06378474 | 0.235  (0.1596) | 0.140768 | 0.314  (0.6651) | 0.63697554 | 0.301  (0.2765) | 0.2757864 | 0.203  (0.1921) | 0.2896165 | 0.225  (0.1883) | 0.2317224 | 0.236  (0.2842) | 0.4057007 |
| Other/Unknown | 1.241  (0.137) | 1.2829E-19 | 1.275  (0.1433) | 5.8584E-19 | 2.629  (0.8164) | 0.00128337 | 1.388  (0.2604) | 9.8261E-08 | 1.213  (0.1694) | 7.979E-13 | 1.133  (0.1638) | 4.6122E-12 | 1.62  (0.2855) | 1.4029E-08 |
| Tumor size | 0.003  (7e-04) | 1.4003E-06 | 0.003  (7e-04) | 6.3175E-06 | 0.001  (0.0092) | 0.92388607 | 0.003  (9e-04) | 0.00159761 | 0.005(  0.0018) | 0.00468469 | 0.004  (0.002) | 0.02653452 | 0.003  (8e-04) | 0.00056374 |
| **Geographical Region (Ref = non-Appalachia)** | | | | | | | | | | | | | | |
| Appalachia | -0.044  (0.1049) | 0.6723506 | -0.043  (0.1072) | 0.6863951 | 0.567  (0.7059) | 0.42187794 | -0.169  (0.1992) | 0.3971759 | -0.089  (0.1263) | 0.4788459 | NA | NA | NA | NA |
| **Histology (Ref = Adenocarcinoma NOS)** | | | | | | | | | | | | | | |
| Papillary Adenocarcinoma | -0.096  (0.2048) | 0.6376371 | -0.03  (0.209) | 0.8840294 | -0.768  (1.288) | 0.5512367 | -0.598  (0.4155) | 0.1500898 | 0.100  (0.2407) | 0.676876 | 0.002  (0.2398) | 0.9925568 | 0.028  (0.4319) | 0.948838 |
| Tubulovillous Adenocarcinoma | -0.651  (0.4259) | 0.1261181 | -0.818  (0.4583) | 0.07412704 | -1.651  (1.7298) | 0.33993434 | -1.692  (0.7761) | 0.02922619 | -0.584  (0.5183) | 0.2597273 | -0.101  (0.5191) | 0.8456566 | -1.971  (0.8295) | 0.01747958 |
| Mucinous Adenocarcinoma | 0.328  (0.1689) | 0.05249475 | 0.347  (0.1721) | 0.04370414 | 1.130  (1.2673) | 0.37242751 | -0.272  (0.365) | 0.4554246 | 0.415  (0.1978) | 0.0360406 | 0.453  (0.1842) | 0.01398821 | -0.277  (0.481) | 0.5641568 |
| Other/Unspecified | -0.004  (0.1314) | 0.9764196 | 0.047  (0.1359) | 0.7315225 | -1.625  (0.6508) | 0.01254258 | -0.450  (0.2844) | 0.1134517 | 0.139  (0.1549) | 0.371271 | 0.042  (0.1656) | 0.8012593 | 0.040  (0.2313) | 0.8631467 |

Footnote: Analyses are based on a retrospective cohort study of colon cancer patients using data from the Kentucky Cancer Registry collected between January 1, 2010, and December 31, 2022_._ Est (SE): Estimated log hazard ratio from the Cox model (standard error), NOS: Not Otherwise Specified, P-val: Adjusted p-value. For a categorical risk factor, a negative estimate means lower hazard for people in the corresponding category compared to the reference, while larger estimates correspond to greater elevation in the hazard for people in the corresponding categories. Thus, positive estimates identify unfavorable categories.
